# Ophthalmic research and publication in India: Where do we stand?

**DOI:** 10.4103/0301-4738.64115

**Published:** 2010

**Authors:** Barun Kumar Nayak

**Affiliations:** P. D. Hinduja National Hospital and Medical Research Center, Veer Savarkar Marg, Mahim, Mumbai - 400 016, India E-mail: editor@ijo.in

Research in the medical field is a dynamic process without which science will be dead. It has more significance now as we are in the era of evidence-based medicine.

The path of research is not without obstacles. Research needs proper infrastructure and sufficient funds apart from a dedicated group of workers. It demands honesty, commitment and time. In our country, the institution of healthcare gets priority over research for obvious reasons. Research is not preferred as a career by the majority. There are many challenges in the development of research in Asian countries which have been well described by Wong *et al*.[1] Firstly, people do not perceive research as a fulltime job whereas the fact is that it cannot be pursued as a part-time hobby. Secondly, the institutions and departmental heads do not give "high priority" to research activities. High-volume clinical work is given more weightage and recognition. Thirdly, the clinician scientists find that the environment in India is not conducive to perform high-quality research. There are many Indian scientists who are engaged in high-quality research abroad. They are well-recognized initially when they return to India, but most of them do not pursue vigorous research as they consider it a "professional suicide" and not as lucrative and remunerative as clinical practice. Lastly, research ethics are not followed to the core. Due consideration is not given to all the ethical aspects of research and publication. These limitations get compounded due to inadequate infrastructure for even basic postgraduate training in many medical colleges in India.[2‐5] These colleges are obviously not conducive for conducting research. Thesis writing is a mandatory requirement for completion of postgraduate training, but the majority performs research without proper planning and supervision. This is partly due to poor understanding of the entire process of research activity.

Investigators have to overcome many challenges while conducting research in India.[6] Inadequate infrastructure and well-trained staff with high attrition rate are major concerns but are generally taken care of by the sponsors of the study. In most of the institutions in India, the doctor-patient ratio is inadequate; hence they are overstressed performing routine work of basic patient care itself. The opportunity to develop special skills (such as counseling research participants, documentation and pharmacovigilance) required for conducting trials is scarce. Clinicians find it hard to get trained in basic clinical research, good clinical practice (GCP) guidelines, regulatory requirements and ethical issues due to lack of time. The basic qualities of good researchers are enthusiasm, integrity and experience. India is so vast that it is but natural to find a lot of variability in standard of care, attitude and infrastructure. Most of the centers participating in trials have a financial interest as the payment made for the conduction of these studies is high by any standard. "Payment for performance" strategy inadvertently gives rise to conflict of interest. Importance of participation in research planning and protocol writing is not understood by all participating investigators, and most of the institutions find it an easy way out when a readymade protocol is handed over to them.

In spite of the limitations discussed earlier, there is a silver lining. In future, up to 65% of food and drug administration (FDA)-regulated trials are expected to be outsourced from the US. India and China have been projected as the favorite destinations for these clinical trials.[6] Thatte *et al*.,[6] have described various reasons for this anticipation. There are many hospitals in the country with state-of-the art facilities. These hospitals have well-trained English-speaking physicians with a huge patient load. India also has a large population of treatment-naïve patients. A good network of accredited labs helps in sufficient enrollment of compliant subjects who can be retained till the end of the study. India does not lag behind in good communication and information technology capabilities which are of great help in these studies. Data entry, analysis, management and finally report writing are now easy to handle. To a large extent the negative perception of trials in the minds of the subjects is fast disappearing due to well-informed subjects. All this helps in building a strong patient base. Nowadays, all trials are managed by clinical research organizers (CROs), who find it quite economical to conduct research in India. It would not be inappropriate to mention that one should be careful and vigilant while signing the clinical research agreement (CRA).[7] It is advisable that the multicentric trial protocol and CRA should also go through the institutional review board (IRB) and ethics committee of the respective participating centers.

Completion of research has no meaning unless it is published in peer-reviewed journals. Dhaliwal *et al*.,[8] have reported that out of 200 free papers presented at the All India Ophthalmic Society Annual Conference (AIOC) 2000, only 33 papers were published in the subsequent seven years in Pubmed-indexed journals. Such a low percentage of publication from completed works shows apathy towards publication by researchers. The other possible cause could be a lack of quality researches presented in AIOC. In another study by Dhaliwal *et al*.,[9] it was concluded that only 30% of theses by postgraduate students culminated in publication from a university medical college in India. Kumaragurupari *et al*.,[10] must be congratulated for a study entitled "A bibliometric study of publications by Indian ophthalmologists and vision researchers, 2001-06" published in this issue of the *Indian Journal of Ophthalmology* (IJO). Their conclusion shows a heartening trend of nearly doubling of publications from 284 in 2001 to 460 in 2006 by Indian researchers. However, 50% of the publications came from only nine major eye hospitals in India. A Pubmed search of publications by Indian authors and institutions in 2008 yielded 16290 results as against 11307 in 2005. Hopefully, the 2009 amendment of the regulations of the Medical Council of India (MCI) for appointment of teachers will provide enough incentive for researchers to publish their completed research.[11]

The scope for research in India is tremendous simply because India is a vast country with a rapidly improving economy. There are many organizations, such as the Indian Council of Medical Research (ICMR), Department of Biotechnology (DBT), Department of Science and Technology (DST) and others who are prepared to fund the appropriate research. ICMR has identified the scope of research in ophthalmology and has appointed coordinators for the various sub-specialties.

There is a need to understand the nuances of research. Researchers should realize the importance of taking IRB/ethics committee approval before starting the study. It is now mandatory to take IRB/ethical clearance for the dissertation done during the Diplomate of the National Board (DNB) course.[12] Many journals do not accept articles for publication if randomized controlled trials are not registered with the trial registry. IJO in association with the P. D. Hinduja National Hospital and Medical Research Center has taken the initiative of conducting regular courses on 'research methodology'. The popularity of this course is an indication of the growing interest in research amongst ophthalmologists. ICMR offers a short-term studentship (STS) program, which is also becoming very popular.[13]

To conclude, India is fast becoming a new destination for multicentric trials. It is time to wake up and educate ourselves with the nitty-gritty of research to reap the entire benefit from this favorable trend. We have a huge potential and we must explore it to its full capacity.
